# Thermochemical Recycling of Oily Sludge by Catalytic Pyrolysis: A Review

**DOI:** 10.1155/2021/1131858

**Published:** 2021-10-31

**Authors:** Xiaojing Di, Haodan Pan, Donghao Li, Hongxiang Hu, Zhiyong Hu, Yulin Yan

**Affiliations:** ^1^College of Petroleum Engineering, Liaoning Petrochemical University, Fushun 113301, China; ^2^CAS Key Laboratory of Nuclear Materials and Safety Assessment, Institute of Metal Research, Chinese Academy of Sciences, Shenyang 110016, China; ^3^The Shale Oil Plant of Fushun Mining Group Co., Ltd., Fushun 113115, China

## Abstract

The main methods of treating oily sludge at home and abroad and the current research status of oily sludge pyrolysis technology are briefly described, and four commonly used catalysts are introduced: metals, metal compounds, molecular sieves, metal-supported molecular sieves, and biomass catalysts for oily sludge. The influence of pyrolysis, the pyrolysis mechanism, and the product composition of oily sludge with the addition of different catalysts are also discussed. Finally, the development direction of preparing new catalysts and the mixed use of multiple catalysts is proposed as a theory to provide for the efficient and reasonable utilization of oily sludge.

## 1. Introduction

### 1.1. Source and Ingredients of Oily Sludge

Crude oil is the blood of industry and an important strategic resource in the development of the national economy. The oil oily sludge is inevitably produced during the storage, transportation, and refining development process of the entire petroleum industry. It is characterized by high oil content, fine particles, high viscosity, and difficulty in dehydration and transportation. The composition of oily sludge is extremely complex, containing high concentrations of total petroleum hydrocarbons, and heavy metals, such as copper, zinc, chromium, mercury, chemical additives, and pathogenic microorganisms. According to research, the oil content of oily sludge is between 10% and 50%, and the water content is between 40% and 90%, accompanied by a certain amount of solids.

The output of oily sludge is increasing year by year. According to statistics, the total amount of oily sludge produced in the world every year reaches 60 million tons [1–3], and with the deep development of most oil fields, the output of oily sludge will continue to increase. According to the source, oily sludge can be divided into oilfield oily sludge (OOS), storage and transportation oily sludge (STOS), refinery oily sludge (ROS), and accidental oily sludge (AOS). Different oily sludges differ in properties. Selecting the corresponding oily sludge treatment method according to its nature is a task that must be completed at present.

### 1.2. Treatment Method

At present, domestic and foreign methods for treating oily sludge include thermochemical cleaning technology, solvent extraction technology, and biological treatment technology.

#### 1.2.1. Thermochemical Cleaning

Thermochemical cleaning technology is a method of adding hot water and chemical reagents to oily sludge to change the properties of the oil phase and liquid phase to extract oil. Most of the thermochemical cleaning technology treats oily sludge incompletely, and it is difficult to meet the demand. Therefore, its method will be combined with biological treatment technology, using thermochemical cleaning as pretreatment to refine crude oil and biological treatment technology to achieve harmless treatment of its products. Duan et al. [4] evaluated the cleaning effect by using the interfacial tension (IFT) method and found that when the mixture ratio of Na_2_CO_3_, AEO-9, and rhamnolipid is 5 : 1 : 0.5, the IFT can be reduced to 0.064 mN/m as low as possible. When the concentration of the mixture is 2 wt%, the cleaning agent aqueous solution/oily sludge is 3 : 1, the cleaning time is 40 minutes, the cleaning temperature is 50°C, and the residues on the cleaned solids are analyzed. The oil is mainly passed through the layer adsorption, where its adsorbed on the solid, and the residue is paraffin and polycyclic aromatic hydrocarbons. Wang et al. [5] achieved the three-phase separation of oil, mud, and water through precipitation and swirling and used gel permeation chromatography (GPC) to characterize the migration of the oil phase. As the cleaning solvent/oil ratio increased from 1 : 2 to 5: 1, the molecular weight of the upper layer is reduced from 1044 to 846, and the solvent is a necessary condition for separating the oil phase. Jing et al. [6] used AEO-9, DBS, Na_2_SiO_3_-9H_2_O, and other surfactant solutions to test the best solution for washing oily sludge. Because Na_2_SiO_3_-9H_2_O is easy to disperse, the residual oil rate is the lowest, only about 1.6%, and the washing effect is the best. Compared with other technologies, chemical cleaning technology has the advantages of simple process, low cost, and high reliability, and most of the solutions can be recycled, which can avoid secondary pollution.

#### 1.2.2. Solvent Extraction Technology

The solvent extraction process is widely used as an operating technology to remove semivolatile and volatile organic compounds from oily sludge. The oily sludge is mixed with the solvent in a certain ratio, and the oil is extracted by the solvent and then recovered by distillation. Commonly used solvents include methyl ethyl ketone (MEK), reformed oil, liquefied petroleum gas (LPGC), hexane, xylene, and other organic solvents, which can be recycled. Due to the large consumption of solvents, high costs, and possible new environmental pollution, in recent years, researchers have devoted themselves to the development of new solvents such as supercritical liquids to replace traditional organic solvents. The extraction process is faster than traditional organic solvents, but when the processing volume is too large, the efficiency drops [7]. Lam et al. [8] summarized recent solvent extraction experiments and found that solvent extraction has the disadvantage of inconvenient recovery of waste oil.

Khan et al. [9] developed supercritical fluid solvents instead of traditional chemical solvents. This technology can remove metal impurities, break emulsions, and complete petroleum extraction without the use of catalysts and hydrogen. The oil recovery rate can reach 78%; though, the quality content is reduced by 98.5%. The RAG layer conversion of supercritical fluids has created a new method in the field of nontraditional crude oil refining. This process is suitable for oil refineries to efficiently extract oil recovery fluid. Hu et al. [10] used solvents to extract the petroleum in the oily sludge and performed thermogravimetric analysis on the oily sludge, the extracted crude oil, and the residual oily sludge. The temperature range of the extracted crude oil was 200-550°C. However, the characteristics of the initial oily sludge still remain, indicating that the extraction is not complete, and there is still value for further development. Compared with other technologies, the solvent extraction method has a higher oil recovery rate, thorough treatment of oily sludge, lower extraction temperature, energy saving, and mild operating conditions, and the extraction agent does not need to be regenerated. It is a clean, economical, and effective oily pollution.

In mud treatment technology, the extractant is volatile and toxic, and the tightness of the equipment is strictly required during operation, which leads to high processing costs. At present, the extraction method is considered to be an efficient method for treating oily sludge. To solve the shortcomings of the above method, it is necessary to pretreat the oily sludge and select a suitable extractant as the goal of indepth requirements shortly.

#### 1.2.3. Biological Treatment Technology

Biological treatment is a process that uses microorganisms to decompose the hydrocarbons in the oily sludge into carbon dioxide and water to achieve harmlessness [11]. Most of the petroleum products in oil oily sludge can be decomposed by microorganisms, and the decomposition rate is mainly affected by the composition of hydrocarbons. There are three types of biological treatment: land cultivation, composting, and bioreactor method. Zare et al. [12] concluded that most of the petroleum substances in the oily sludge can be decomposed by microorganisms, and the decomposition speed varies with the types of hydrocarbons. Alkanes are the most easily decomposable hydrocarbons; in contrast to asphaltenes, microorganisms can directly decompose without additional conditions, but the natural decomposition by microorganisms is very slow. Ji et al. [13] optimized the activity and stability of the formate dehydrogenase gene Cbfdh by using Box-Behnken, and the Cbfdh activity reached 12.2. Using Cbfdh to degrade 10% (w/w) oily sludge for 12 hours; the degradation rate could reach 35.6%. CbFDH makes full use of lactose in Escherichia coli BL21 to accelerate crude oil degradation, and its Cbfdh efficiency and cost are better than current microbial strains. Compared with other methods, the biological treatment method is not expensive, has low safety pollution, and has strong transferability.

#### 1.2.4. Heat Treatment Technology

Traditional oily sludge treatment methods are mainly landfilling, composting, and incineration. The landfill method is to fill, cover, and compact the oily sludge into a prepared pit, make it undergo biological, physical, and chemical changes, decompose organic matter, and achieve the purpose of reduction and harmlessness. Landfilling has low cost and easy operation and is generally regarded as a general solution for oily sludge treatment. Compost can decompose organic materials into organic fertilizer for agriculture. Hu et al. [14] summarized the disadvantages of the composting method, which covers a large area and is easily affected by natural conditions such as rainfall and temperature. Incineration is considered to be the most effective method to reduce the volume of oily sludge, which can significantly reduce the amount of waste. Gong et al. [15] burned oily sludge in a tube furnace and used the software FactSage to calculate the thermodynamic balance of the process. As the incineration temperature increased from 800°C to 1000°C, Cr, Pb, Cu, Zn, Ni, etc, the release rate of heavy metals gradually increased. When the temperature exceeded 1000°C and the excess air ratio increased from 1.0 to 1.4, the release rates of Cr, Pb, Cu, Zn, and Ni decreased first and then stabilized. Guo et al. [16] found that incineration decomposes the oil and other organic pollutants in oily sludge into small molecules, such as H2O and CO2. Zhou [17] concluded through experiments that the incineration temperature should be controlled above 850°C to avoid the production of dioxins. Although heat treatment has certain advantages, there are certain problems in terms of oily sludge treatment effect, cost, applicability, and secondary pollution.

Traditional oily sludge treatment methods include landfill, composting, and incineration, which occupy a large area, are costly, and are likely to cause secondary pollution. Some emerging treatment methods, such as solvent extraction technology and biological treatment technology, will not cause secondary pollution, but there are disadvantages such as high cost and long cycle. Pyrolysis is therefore gradually being valued and widely used due to its fast processing, good effect, and availability of residues.

## 2. Research Progress of Catalytic Pyrolysis of Oily Sludge

### 2.1. Pyrolysis and Catalytic Pyrolysis

**Figure 1:**
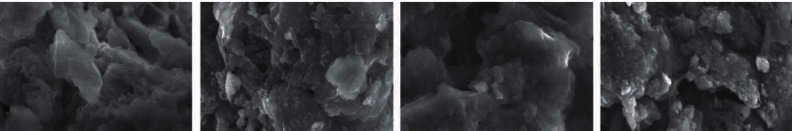
Changes in the apparent morphology of raw oily sludge and oily sludge after pyrolysis at 450°C, 750°C, and 950°C for 0 weeks [18].

**Figure 2:**
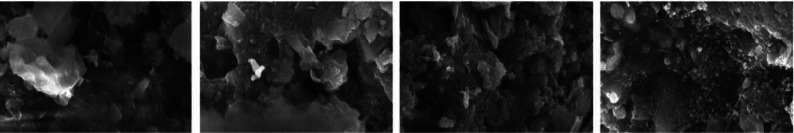
Changes in the apparent morphology of the original oily sludge and oily sludge after pyrolysis at 450, 750, and 950°C for 2 weeks [18].

**Figure 3:**
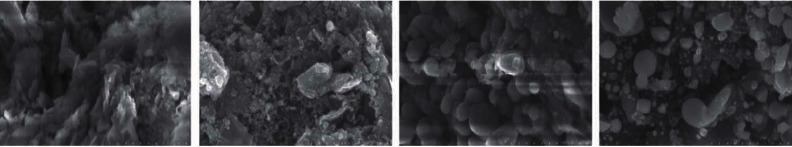
Changes in the apparent morphology of raw oily sludge and oily sludge after pyrolysis at 450°C, 750°C, and 950°C for 4 weeks [18].

Pyrolysis technology means that the oily sludge is heated to a certain temperature under anaerobic conditions to separate the hydrocarbons and recover them through phase separation. The recovered gas phase is methane, carbon dioxide, etc., the liquid phase is mainly fuel and water at room temperature, and the solid phase is inorganic minerals and carbon residue. In this process, organic components are transformed into pyrolysis oil, gas products, and carbonaceous residues. After pyrolysis, the oily sludge is gradually mineralized, as shown in Figures 1–3 [18]. The thermal conversion process of oily sludge is divided into two stages: first, the evaporation stage when the temperature is lower than 350°C, low-boiling light hydrocarbons volatilize from the oily sludge. The second stage is the parallel-sequential reaction stage, and when the temperature exceeds 350°C, heavy oil will start to crack. Hydrocarbons will generate free radicals due to thermal activation at around 400°C, and a series of free radical reactions will occur. On the one hand, it will proceed towards the cracking of small-molecule hydrocarbons while it proceed towards the condensation direction of coking and finally produce oil, water, noncondensable gas, and coke [19]. In comparison, the pyrolysis method has the advantages of faster processing speed, more thorough oily sludge treatment, and recyclable oil and gas and residues. However, it also has large investments, high energy consumption, high equipment requirements, and more complicated operations. Improper disposal is prone to defects such as secondary pollution and potential safety hazards.

In response to the problems of pyrolysis of oily sludge, researchers have focused on further improving the yield and quality of pyrolysis products by adjusting the process conditions of the pyrolysis process. Gong et al. [20] studied the pyrolysis temperature of oil oily sludge in a tube furnace and the influence of different atmospheres (N2/CO2) on the pyrolysis behavior of oily sludge. CO_2_ can promote the pyrolysis of OS and reduce the coke yield. As the pyrolysis temperature increases, the light fraction (gasoline, diesel) in the oil decreases, the heavy fraction also increases, while the content of CH_4_, C_2_H_4_, C_4-6_, and C_6+_ increases, and the content of C_2_H_6_, C_3_H_8_, and C_3_H_6_ decreases. Lin et al. [21] studied the copyrolysis of oily sludge and rice husk at a weight ratio of 2 : 1, the increase in the content of SARA (saturates, aromatics, resins, asphaltenes) saturates and aromatics, and the reduction of heavy fractions and improve the quality of oil solution. The concentration of chain hydrocarbons increased, and the content of oxygenated compounds decreased by 46-93%. As the synergistic reaction promotes the secondary reaction, pyrolysis tends to produce more H_2_, CO, and C_1_-C_2_ hydrocarbons.

Regarding the adjustment of pyrolysis process conditions to promote pyrolysis of oily sludge, such as catalytic pyrolysis, it has shown great development potential and research prospects due to its high treatment efficiency and low pollution. Adding a catalyst in the pyrolysis process can have a positive effect on the pyrolysis products of oily sludge, that is, the oil and gas yields increase, and the oil quality is improved, while the residue reduces [22, 23]. It can also change the pyrolysis reaction conditions to make the time required for pyrolysis shorter, and the temperature is lowered. For the same oily sludge, different catalysts are pyrolyzed and catalyzed, and the products after pyrolysis have different degrees of influence. Therefore, it is very important to determine the results of the experiment and choose the appropriate catalyst.

### 2.2. Research Progress of Catalysts for the Catalytic Pyrolysis of Oily Sludge

Common catalysts for pyrolysis of oily sludge include: metals, metal compounds, molecular sieves, and biomass.

#### 2.2.1. Metal

Metal element catalysts are mainly transitioned metal elements, including Ni, Fe, Cu, and Al, and are generally loaded on a carrier to improve the pyrolysis efficiency of oily sludge [24, 25]. Metal catalysts can increase the generation of pyrolysis gas [26].

Li et al. [27] mixed oily sludge and steel slag for reaction, which can recover oil and gas and is accompanied by iron generation. As the two react together, the steel slag undergoes a reduction reaction at high temperatures to produce iron. The reduced iron acts as a catalyst for the pyrolysis of oily sludge. Compared with the pyrolysis of oily sludge alone, the production of CO2 and H2 is significantly increased. Lin et al. [28] proposed an innovative method to prepare Fe-char from oil oily sludge and apply it to the catalytic cracking of heavy oil in oily sludge. The conversion efficiency of char900 formed at 900°C is higher than that of char600 formed at 600°C. By using char900, the H_2_ and CO yields are significantly improved. The oil conversion rate of using char900 at 800°C can reach 95.8%, Fe°. It plays an important role in promoting hydrocarbon reforming and water gas shift (WGS) reactions. Song et al. [29] innovated a method of combining oil oily sludge and steel slag to improve the quality of the oil. Thermogravimetric analysis (TG) was used to characterize the characteristics of pyrolysis oil, and the effect of steel slag on the product H_2_ during the pyrolysis of oily sludge was analyzed. The addition of steel slag increases the weight loss rate of oil sludge and promotes the production of H_2_ and CH_4_. When 15% steel slag is added, the H_2_ yield increases from 26.43% to 30.41%, and the CH_4_ yield increases from 34.65% to 43.38%. The pyrolysis of oily sludge is 3 times. At the same time, it is feasible to use a magnetic separator to separate steel slag and oily sludge after the experiment, and this scheme has a certain potential. Metals play a positive role in the pyrolysis of oily sludge, but due to high cost, it is therefore necessary to choose this type of catalyst based on actual conditions.

#### 2.2.2. Metal Compounds

The metal compound is a catalyst mainly composed of light metals, such as compounds of Al, Na, Ca, K, etc. Metal oxides have excellent redox characteristics and high catalytic activity [26, 30]. Compared with ordinary zeolite molecular sieve catalysts, metal oxide catalysts are easy to obtain; so, metal oxide catalysts have a high-cost performance.

Kwon et al. [22] found that adding CaCO3 to the oily sludge cannot only increase the CO generation rate but also reduce the yield of polycyclic aromatic hydrocarbons from 36.6% to 29.6%. The CO2 generated by the heating of CaCO3 enhances the thermal cracking of volatile organic carbon during the pyrolysis of OS and provides a source of C and O, thereby increasing the yield of CO. The addition of CaCO3 can improve the efficiency of converting solids into gases, thereby reducing the production of harmful substances. Lin et al. [31] explored the effect of KOH on the pyrolysis of different oily sludges and used gel permeation chromatography (GPC), rheometer, and gas chromatography-mass spectrometry (GC-MS) to characterize pyrolysis oil products. When added, oil yield and oil viscosity are reduced, and gas and solid products and calorific value are increased. KOH can promote the formation of compounds in pyrolysis oil and inhibit the formation of asphalt. KOH promotes the improvement of oil quality with low molecular weight, low viscosity, and high calorific value. Huang et al. [32] studied the effect of adding Fe2O3 on the composition and content of solid-liquid gas produced by oily sludge pyrolysis. Its Fe2O3 increased the gas produced from 8.69 wt% to 11.62 wt%, and the oil increased from 32.54 wt% to 38.74 wt%. The generated char is reduced from 58.77 wt% to 49.64 wt%. This catalyst accelerates the formation of gas, oil, and char, but does not affect the components in the oil. Sun et al. [33] proposed a pyrolysis scheme of fully mixed biomass-coated alumina and pyrolyzed oily sludge and studied the effect of temperature and alumina on pyrolysis. The yield and component distribution of noncondensable gas products are more significantly affected by temperature. When the mass ratio of alumina to oily sludge at 500°C is 1 : 35, the maximum liquid production is 48.44 wt.%. As the temperature increases, the liquid product undergoes secondary cracking, and CO_2_ and H_2_ increase. In summary, compared with metal catalysts, metal compound catalysts cannot only increase the yield of pyrolysis gas but also increase the yield of hydrocarbons and their pyrolysis oil compounds. Such catalysts are easy to obtain, and different metal compounds have different effects on the pyrolysis of oily sludge, which need to be selected according to specific needs.

#### 2.2.3. Molecular Sieves and Metal-Supported Molecular Sieve Catalysts

Molecular sieves are a kind of aluminosilicate crystals with regular microporous structure [34], and they are widely used in X type, Y type, ZSM-5 type, MCM-41 type, and so on [26, 35].

Molecular sieves are widely used in heterogeneous catalysis and as catalyst carriers due to their advantages in pores and large specific surface area. However, many molecular sieves are relatively weak in acidity with low surface density, resulting in low catalytic activity. Therefore, the application of mesoporous molecular sieves to catalytic reactions is not ideal. To improve its acidity and catalytic performance, domestic and foreign researchers have used metals or metals. Oxides and other compounds with catalytic effects are introduced into the molecular sieve, which not only takes advantage of the pore advantage of the molecular sieve but also takes advantage of the special properties such as the high activity of the oxide. Jia et al. [36] prepared Fe/Al pillared bentonites with different Fe/Al mass ratios as oily sludge pyrolysis catalysts and used X-ray diffraction (XRD), N2 adsorption, and NH3-TPD to characterize their pyrolysis products. As the ratio of Fe/Al increases, the oil yield first increases and then decreases. When Fe/Al is 2.0 wt%, the oil yield reaches the maximum with a value of 52.46%. This catalyst can not only increase the oil yield but also increase the formation of CH4. Yu et al. [37] used the impregnation method to synthesize molecular sieve Al-MCM-41 and studied the influence of the catalyst concentration on the pyrolysis of oil oily sludge. As the catalyst concentration increases from 0.5% to 1%, the pyrolysis oil recovery rate increases significantly, reaching the maximum value of 83.48%. After that, the recovered oil did not change much with the increase in catalyst concentration. Lin et al. [38] used HZSM-5 and Zn/HZSM-5 to catalytically pyrolyze oily sludge in a two-stage tubular fixed-bed reactor to recover aromatic hydrocarbons. Through XRD, Fourier transform infrared (FTIR), N_2_ adsorption, and NH_3_-TPD characterize the physical and chemical properties of HZSM-5 zeolite before and after Zn loading. Gas chromatography-mass spectrometry was used to analyze the effects of different residence times and modified HZSM-5 on pyrolysis products. As the residence time increased from 1 second to 7.6 seconds, the total aromatics yield also increased from 48.7% to 92.2%. When the residence time is 1.9 seconds, the catalytic effect of Zn/HZSM-5 on the pyrolysis of oily sludge was studied. The incorporation of 3% Zn increased the total aromatic products from 58.7% to 81.0%, and the H_2_ and CO_2_ yields were significantly improved. In summary, compared to other catalysts, the catalytic effect of supported catalysts is more significant. Compared with ordinary molecular sieves, the catalytic effect of molecular sieves loaded with other catalysts is doubled, and the addition of metals or metal oxides further enhances the catalytic effect of molecular sieves. However, the disadvantage is that the price is high, there is a problem of coking during the reaction, and the temperature of the catalytic pyrolysis reaction is lowered but still above 400°C. How to further improve the low-temperature catalytic efficiency of molecular sieve catalysts can be regarded as the next research goal, and appropriate reaction processes should be selected to prolong the service life of the catalyst, or regeneration treatment should be carried out to improve its use efficiency.

#### 2.2.4. Biomass

The essence of copyrolysis of oily sludge and biomass is to catalyze and strengthen the pyrolysis of oily sludge, enhance the rate of oil and gas recovery, and improve the oil and gas quality. Common biomass catalysts include rice husks, walnut husks, wood chips, and apricot husks [39].

Zhao et al. [40] studied the effects of rice husks, walnut husks, wood chips, and apricot husks on the pyrolysis of oily sludge. As the biomass increases, its dehydration efficiency increases, and the oil recovery rate also increases. The main reason is that biomass can eliminate uneven heat transfer and provide the heat required for pyrolysis. After comparing the pyrolysis products, it is found that the oil recovery rate is sawdust>rice husk>apricot husk>walnut husk. Lin et al. [21] studied the copyrolysis of oily sludge and rice husk and performed SARA and gas chromatography/mass spectrometry tests on the results. SARA concluded that the interaction between the two increases the content of saturated hydrocarbons and aromatics, resulting in a reduction in heavy fractions and an increase in oil quality. Through the gas chromatography/mass spectrometry analysis of the experiment, it is found that the interaction increases the concentration of chain hydrocarbons, CO and C_1_-C_2_ hydrocarbons, and H2 (from 22.2% to 55.6%), but reduces H_2_S. It is mainly attributed to the ash content of biomass and the catalysis of alkali metals. Jin et al. [41, 42] mixed pyrolysis of bamboo sawdust and oily sludge at 400-600°C. Copyrolysis reduced carbon production. In addition, the two copyrolysis promoted the conversion of toxic metals into a more stable state at 600°C. After copyrolysis, Cu and Zn in the stable state of the residue are 3.2 times the pyrolysis of the oily sludge alone. Compared with other catalysts, this catalyst is mostly domestic wastes; so, it is cheap, and conducive for the formation of pyrolysis gas, and can transform potentially toxic metals into a more stable state. Therefore, it is suitable for treating metal-polluted oilfields: a safe and feasible way.

## 3. Comparison of Different Types of Catalysts

The advantages and disadvantages of different types of catalysts and suggestions are shown in Table 1. The adaptability, advantages, and disadvantages of different types of catalysts are different, and suitable catalysts should be selected according to the treatment object, target product, and cost requirements.

## 4. Prospects for Catalytic Pyrolysis Technology of Oily Sludge

The composition of oily sludge is very complex. If it is not treated in time, it will not only cause different levels of environmental pollution but also bring huge economic losses. Therefore, it is urgent to find a harmless treatment method for oily sludge. Suggestions are given below:

1. The main catalysts for oily sludge pyrolysis include metals and their compounds, molecular sieves, and biomass. Various catalysts have different effects due to their different objectives. For the selection of catalysts for experiments, suitable catalysts should be selected according to experimental requirements, results, and process conditions
2. Focus on studying the mechanism of catalysts, develop environmentally friendly and efficient catalysts, and use lower cost and energy consumption to obtain higher quality target products
3. Indepth study of waste recovery and utilization to prepare catalysts to realize energy recycling. Carry out research on the copyrolysis of oily sludge with other biomass and waste, and the two sides have a synergistic effect to promote the reaction and increase the target yield
4. According to the nature of oily sludge emission standards and the comprehensive requirements of environmental protection and economy, there is a need to explore the combined use of multiple catalysts to achieve good catalytic effects

## Figures and Tables

**Table 1:** The advantages and disadvantages of different types of catalysts and suggestions [26].

Catalyst type	Advantage	Shortcoming	Suggest
Metal	Good effect	Expensive, causing secondary pollution	Use according to actual conditions, and use metal waste when appropriate
Metal compound	Variety and easy access	Different metal oxides have different effects on the pyrolysis of oily sludge; so, it is necessary to find a suitable metal oxide	Exploring the metal compounds required by the corresponding oily sludge
Molecular sieve	Not only can be used as a catalyst, but also other catalysts can be supported, so that the catalytic effect is better, and the reaction system is not required.	High price, prone to coking, high pyrolysis temperature	Explore suitable process conditions and carry out catalyst regeneration treatment to extend catalyst life
Biomass	It is not easy to cause secondary pollution, the price is cheap, and the two sides have a coordinated reaction, which promotes a more comprehensive pyrolysis	It is necessary to find suitable biomass for synergistic reaction. Some biomass not only does not promote the reaction but also inhibits the reaction from proceeding.	Explore the biomass that is conducive to the pyrolysis of different oily sludges

## Data Availability

The authors confirm that the data supporting the findings of this study are available within the article.
